# Unrelated umbilical cord blood can improve the prognosis of haploidentical hematopoietic stem cell transplantation

**DOI:** 10.1186/s13287-022-03170-x

**Published:** 2022-09-24

**Authors:** Ying Yang, Ming Zhang, Mengqi Li, Yingchun Li, Wei Yang, Zhuogang Liu, Hongtao Wang

**Affiliations:** Shengjing Hospital of China Medical University, Shenyang, China

**Keywords:** Haploidentical hematopoietic stem cell transplantation, Unrelated umbilical cord blood, Graft versus leukemia, Graft versus host disease, Prognosis

## Abstract

**Background:**

Haploidentical hematopoietic stem cell transplantation (haplo-HSCT) is widely used as a curative treatment strategy for most types of hematological diseases. However, strategies for enhancing the graft versus leukemia (GVL) effect without aggravating the graft versus host disease (GVHD) effect are still being pursued.

**Methods:**

A retrospective cohort study was performed to compare the outcomes between combined unrelated umbilical cord blood (UCB-haplo HSCT) and haplo HSCT.

**Results:**

The results showed that neither acute GVHD (aGVHD) nor chronic GVHD (cGVHD) was increased in the UCB-haplo HSCT group, and the engraftment and infection rates were similar between the two groups. However, overall survival and progression-free survival were significantly improved, while transplantation-related mortality and relapse were significantly decreased in the UCB-haplo HSCT group by both univariate and multivariate analyses.

**Conclusion:**

Our results indicated that the addition of a UCB unit could improve the prognosis of haplo-HSCT and enhance the GVL effect without increasing the incidence of GVHD.

***Trial registration*:**

The cohort study was retrospectively registered at https://www.chictr.org.cn as ChiCTR2100046681.

## Introduction

Allogeneic hematopoietic stem cell transplantation (allo-HSCT) offers a curative treatment strategy for most types of hematological diseases [1, 2]. Haploidentical HSCT (haplo-HSCT) is now widely used, which allows nearly everyone to have suitable donors. With the improvement of the conditioning regimen and graft versus host disease (GVHD) prophylaxis (Beijing protocol), haplo-HSCT has achieved outcomes comparable to those of human leukocyte antigen (HLA)-matched identical sibling HSCT [3]. Cho BS et al. confirmed this conclusion, and their results showed that the 3-year overall survival (OS) rates for HLA-matched-HSCT and haplo-HSCT were 57% [95% confidence interval (CI) 42–69%] and 73% (95% CI 59–83%) in acute myeloid leukemia (AML) patients, respectively [1]. The results of the Beijing protocol also showed the benefit of haplo-HSCT in leukemia, myelodysplastic syndrome (MDS) and aplastic anemia (AA) [4–6]. Despite great improvements achieved in haplo-HSCT, strategies for enhancing the graft versus leukemia (GVL) effect without aggravating the GVHD effect are still being pursued [7].

A previous study combined an unrelated umbilical cord blood (UCB) unit with haplo-HSCT, which reduced the relapse rate of recurrent and refractory acute leukemia [8], indicating that additional UCB infusion played an important role in haplo-HSCT. However, whether unrelated UCB could improve outcomes in patients with different statuses of hematologic malignancy and AA is still unknown. Thus, the cohort study was designed to compare the outcomes of patients between UCB-haplo HSCT and haplo HSCT. The primary endpoints were GVL effects [including OS, progression-free survival (PFS), relapse rate and transplantation-related mortality (TRM)] and GVHD incidence. The secondary endpoints were engraftment and infections. The study was registered at https://www.chictr.org.cn as ChiCTR2100046681.

## Patients and methods

### Patient eligibility

**Fig. 1:**
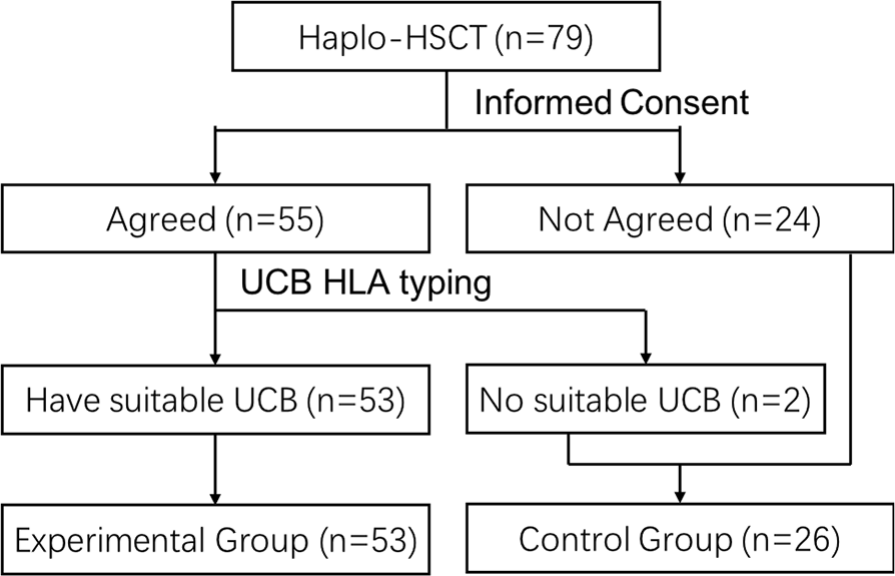
Flow diagram of participant selection and exclusion

Patients who were eligible to receive haplo-HSCT between April 2016 and October 2020 were enrolled in this retrospective cohort study. All the patients were screened and grouped as shown in Fig. 1. The protocols were approved by the institutional review board of Shengjing Hospital of China Medical University. Informed consent for treatment was obtained from all the patients and their donors.

### HLA typing and donor selection

HLA typing was detected by high-resolution DNA techniques for HLA-A, HLA-B, HLA-C, HLA-DQB1 and HLA-DRB1. Donors were selected from family members who shared only one HLA haplotype with the patient. Technically, male donors (especially fathers or sons) were selected. Being a female, mother or second-degree donor was not an ideal choice. In addition, the physical status and willingness of donors were also important matters that physicians should consider.

### UCB selection

The selection of UCB units was based on HLA typing results and dose assessment before cell freezing. The UCB units were obtained from Liaoning and Shandong UCB banks certified by the Ministry of Health. All units were qualified clinical grade, normal in volume with depleted red blood cells, and transferred by cold-chain transportation. The selection strategy was as follows: First, HLA matching with patients required 3-6/6 with high-resolution HLA typing for HLA-A, HLA-B and HLA-DRB1. Second, minimum HLA matching required 7-9/10 high-resolution HLA typing with patients for HLA-A, HLA-B, HLA-C, HLA-DQB1 and HLA-DRB1. Third, blood type and sex were compared between UCB units and recipients, and UCB cell numbers were evaluated and considered.

### Transplantation regimen

The transplantation regimen and GVHD prophylaxis strategy for malignant hematologic disease and AA were performed according to “Beijing protocols” reported previously, which includes the transplantation of granulocyte colony-stimulating factor (G-CSF)-mobilized peripheral blood stem cells (G-PBSCs) and G-CSF-primed bone marrow (G-BM) from HLA-mismatched/ haploidentical related donors without in vitro T-cell depletion. [9, 10]. The conditioning therapy for malignant hematologic disease consisted of cytarabine (Ara-C, 4 g/m^2^/day) administered intravenously on days − 10 to − 9, busulfan (BU, 3.2 mg/kg/day) administered intravenously on days − 8 to − 6, cyclophosphamide (CTX, 1.8 g/m^2^/day) administered intravenously on days − 5 to − 4, semustine (250 mg/m^2^) administered orally on day − 3 and anti-thymocyte globulin (ATG, 2.5 mg/kg/day, rabbit) administered intravenously on days − 5 to − 2. The conditioning regime for AA consisted of BU (3.2 mg/kg/day) administered intravenously on days − 7 and − 6, CTX (50 mg/kg/day) administered intravenously on days − 5 to − 2, and ATG (2.5 mg/kg/day, rabbit) administered intravenously on days − 5 to − 2. All transplantation recipients were given cyclosporin A (CsA), mycophenolate mofetil (MMF) and short-term methotrexate (MTX) for GVHD prophylaxis. The regimen of prevention, monitoring, intervention and treatment of relapse was in accordance with previous studies [11].

All patients received G-BM and G-PBSCs from haploidentical donors on Days 0 and 1, respectively. In addition to haploid grafts, unrelated UCB units in the UCB-haplo group were infused on day 0 at least 4 h before haploidentical bone marrow infusion.

Implant chimerism monitoring was determined by short tandem repeat polymerase chain reaction (STR-PCR) among recipient, donor and unrelated UCB.

### Definition and assessments

Neutrophil engraftment was defined as the first day when the absolute neutrophil count was > 0.5 × 10^9^/L for 3 consecutive days. Platelet engraftment was defined as a platelet count > 20 × 109/L for 7 consecutive days without platelet transfusion. Criteria for response in AA: (a) none: still fulfil severe disease criteria; (b) partial: transfusion independent and no longer meet criteria for severe disease; and (c) complete: hemoglobin concentration normal for age and gender; ANC > 1.5 × 10^9^/L and platelet count > 150 × 10^9^/L. The diagnosis of GVHD was in accordance with the common international criteria [12–14]. CMV-related disease was defined according to reported criteria [15]. Overall survival (OS) time was defined as the time from hematopoietic stem cell transfusion to death by any cause. Progression-free survival (PFS) time was defined as the time from hematopoietic stem cell transfusion to disease progression or death. Relapse was defined by morphologic evidence of disease in peripheral blood, bone marrow and extramedullary sites or by the recurrence and sustained presence of pretransplantation chromosomal abnormalities. For AA, the loss of complete response was defined as relapse. Transplant-related mortality (TRM) was defined as death due to causes unrelated to the underlying disease.

### Statistical analysis

Data were censored at the time of death or the last available follow-up on July 01, 2021. Data were collected from the institutional database and verified by the primary investigators and staff of the HSCT team.

## Result

### Patient characteristics

A total of 79 patients who were eligible to receive HSCT were enrolled in the study. All patients were suggested to receive an additional third-party UCB infusion, and 24 patients did not agree to use UCB. Fifty-five patients were enrolled to search the suitable UCB units as a previous scheme in Liaoning and Shandong UCB banks. However, two patients had no suitable UCB, and 53 patients had suitable UCB were recruited into the experimental group (UCB-haplo group) and provided informed consent for UCB treatment. The left 26 patients were recruited into the control group (haplo group) (Fig. 1). The patients’ characteristics are given in Table 1.

**Table 1:** Patient characteristics

Parameter	UCB-haplo group (*n*, percent %)	Haplo group (*n*, percent %)
Age at HSCT, years		
< 20	15/53 (28.30%)	9/26 (34.62%)
20–40	27/53 (50.94%)	11/26 (42.31%)
> 40	11/53 (20.75%)	6/26 (23.08%)
Gender		
Male	27/53 (50.94%)	14/26 (53.85%)
Female	26/53 (49.06%)	12/26 (46.15%)
Disease diagnosis		
AA	15/53 (28.30%)	4/26 (15.38%)
MDS	11/53 (20.75%)	6/26 (23.08%)
AML	15/53 (28.30%)	9/26 (34.62%)
ALL	9/53 (16.98%)	5/26 (19.23%)
Others	3/53 (5.66%)	2/26 (7.69%)
Disease status		
CR1	18/53 (33.96%)	7/26 (26.92%)
CR2	2/53 (3.77%)	2/26 (7.69%)
MRD positive	2/53 (3.77%)	1/26 (3.85%)
PR	0/53 (0.00%)	5/26 (19.23%)
NR	16/53 (30.19%)	7/26 (26.92%)
Donor recipient relationship		
Female-male	4/53 (7.55%)	4/26 (15.38%)
Second-degree donor	4/53 (7.55%)	0/26 (0.00%)
Others	45/53 (84.90%)	22/26 (84.62%)
Mismatched HLA		
3 loci	34/53 (64.15%)	13/26 (50.00%)
2 loci	9/53 (16.98%)	5/26 (19.23%)
0–1 locus	10/53 (18.87%)	8/26 (30.77%)
ABO match		
Matched	29/53 (54.72%)	13/26 (50.00%)
Minor mismatched	10/53 (18.87%)	4/26 (15.38%)
Major mismatched	10/53 (18.86%)	7/26 (26.92%)
Major and minor mismatched	4/53 (7.55%)	2/26 (7.69%)
EBMT risk score		
0–1 point	17/53 (32.08%)	4/26 (15.38%)
2–3 points	19/53 (35.85%)	9/26 (34.62%)
4–6 points	17/53 (32.08%)	13/26 (50.00%)

HSCT, hematopoietic stem cell transplantation; UCB-Haplo Group, haploidentical HSCT with an unrelated umbilical cord blood infusion; AA, aplastic anemia; MDS, myelodysplastic syndrome; AML, acute myeloid leukemia; ALL, acute lymphoid leukemia; CR, complete remission; MRD, minimal residual lesion; PR, partial remission; NR, non-remission; HLA, human leukocyte antigen; EBMT, the European group for blood and marrow transplantation

### UCB and haploid graft characteristics

Following the previously described protocol, UCB units were chosen by the same physician and rechecked by another transplantation group physician. The characteristics of UCB units and haploid grafts are listed in Table 2. In addition, the quantities of MNC and CD34^+^ cells in haploid grafts were not significantly different between the UCB-haplo group and the haplo group. For HLA matching between the UCB unit and recipient, 10 units were 9/10 matched, 26 units were 8/10 matched, and 17 units were 7/10 matched. Before UCB infusion, an anti-allergy regimen was performed. There was no obvious transfusion reaction observed.

**Table 2:** Grafts data

	UCB-haplo group	Haplo group	*P* value
Haploid grafts			
MNC (10^8^/kg)	5.96 ± 2.74	6.07 ± 2.35	0.865
CD34^+^ cells (10^6^/kg)	5.31 ± 2.91	6.07 ± 2.35	0.633
UCB units			
MNC (10^6^/kg)	8.81 ± 4.42		
CD34^+^ cells (10^5^/kg)	0.75 ± 0.74		

UCB-Haplo Group, haploidentical hematopoietic stem cell transplantation with an unrelated umbilical cord blood infusion; MNC, mononuclear cell

### Engraftment

All surviving patients underwent chimerism analysis at Day 30 after HSCT, and they all achieved full donor chimerism. Chimerism analyses in these patients were continued regularly until disease relapse. During follow-up, in UCB-haplo group, there were two patients experienced mixed chimerism (unrelated UCB and haplo-identical donor). One patient was found to have mixed chimerism at the six-month visit with a normal range of routine blood tests and died at Day 235 after HSCT because of a serious fungal infection. The other patient was found to have mixed chimerism at Day 60 after HSCT and turned to full donor chimerism at Day 90 after HSCT.

The day of neutrophil and platelet engraftment was not significantly different between the UCB-haplo group and the haplo group. The median day of neutrophil engraftment was at day 12 (range, 10–24) and day 13 (range, 10–42) for the UCB-haplo group and haplo group, respectively (*P* = 0.349). The cumulative incidence of neutrophil engraftment on day 30 was 100% in the UCB-haplo group and 96% (95% CI 72.7–99.4%) in the haplo group (*P* = 0.52). Meanwhile, the median day of platelet engraftment was at day 14 (range, 9–69) and day 13 (range, 8–96) for the UCB-haplo group and haplo group, respectively (*P* = 0.974). The cumulative incidence of platelet engraftment on day 100 was 100% in both the UCB-haplo group and haplo group (*P* = 0.55).

### GVHD

Both aGVHD and cGVHD were considered in the present study. In the UCB-haplo group, the 100-day cumulative incidences of grade II-IV aGVHD and grade III-IV aGVHD were 24.53% (95% CI 12.01–35.27%) and 5.67% (95% CI 0–11.68%), respectively. However, in the haplo group, the 100-day cumulative incidences of grade II–IV aGVHD and grade III–IV aGVHD were 15.38% (95% CI 0.31–28.18%) and 7.69% (95% CI 0–17.39%), respectively. There was no significant difference between the two groups (*P* = 0.36 and 0.73, respectively). The 1-year cumulative incidence rates of cGVHD in both the UCB-haplo group and the haplo group were 30.19% (95% CI 16.67–41.52%) and 38.46% (95% CI 16.61–54.59%), respectively. There was also no significant difference between the two groups (*P* = 0.45).

### CMV and EBV infection

The 100-day cumulative incidence of CMV viremia was 47.17% (95% CI 31.87–59.04%) in the UCB-haplo group versus 50.00% (95% CI 26.56–65.96%) in the haplo group (*P* = 0.78).

The 100-day cumulative incidence of EBV viremia was 39.62% (95% CI 24.9–51.45%) in the UCB-haplo group versus 19.23% (95% CI 2.57–33.04%) in the haplo group (*P* = 0.062).

### OS, PFS, TRM and relapse rate

**Fig. 2:**
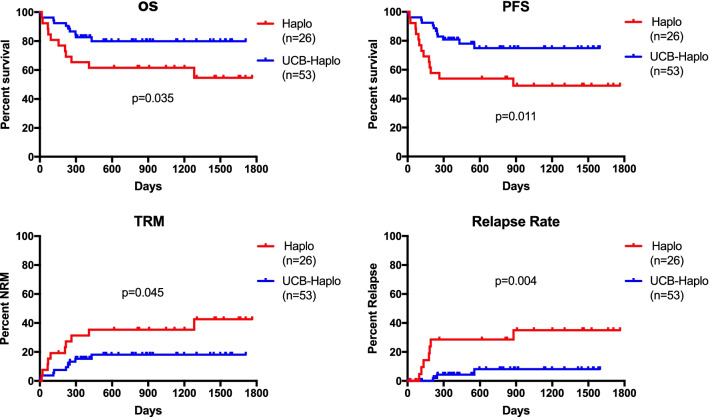
Overall survival, progression-free survival, transplantation-related mortality and relapse rate after haplo and UCB-Haplo HSCT

The probability of OS in the UCB-haplo and haplo groups was 79.92% (95% CI 69.39–92.04%) and 61.54% (95% CI 45.41–83.39%), respectively (*P* = 0.035). (Fig. 2) PFS in the UCB-haplo and haplo groups was 74.92% (95% CI 63.25–88.74%) and 53.85% (95% CI 37.72–76.86%), respectively (*P* = 0.011). (Fig. 2).

The cumulative incidence of TRM was 18.13% (95% CI 6.47–28.34%) and 35.38% (95% CI 13.78–51.58%) in the UCB-haplo and haplo groups, respectively (*P* = 0.045). (Fig. 2) The cumulative incidence of relapse was 8.10% (95% CI 0.00–16.87%) and 28.57% (95% CI 6.38–45.50%) in the UCB-haplo and haplo groups, respectively (*P* = 0.004). (Fig. 2).

The probability of PFS excluding AA in the UCB-haplo and haplo groups was 66.72% (95% CI 51.76–85.99%) and 50.00% (95% CI 32.92–75.94%), respectively (*P* = 0.049). The cumulative incidence of relapse excluding AA patients was 12.36% (95% CI 0.00–25.39%) and 29.41% (95% CI 4.06–48.06%) in the UCB-haplo and haplo groups, respectively (*P* = 0.029).

### Univariate analysis

Univariate analysis was performed to determine the factors for predicting survival and relapse. The basic characteristics of patients and donors were used to identify predictive factors, and unrelated UCB, GVHD and infection were also included. The results showed that older age, poor disease status, high EBMT risk score, accompanying cGVHD, infection and the absence of a UCB unit were associated with poor OS and PFS as measured by univariate analysis (*P* < 0.10, shown in Table 3). Per univariate analysis, disease status, EBMT risk score, cGVHD, infection and the combination of UCB were also related to TRM (*P* < 0.10, shown in Table 3). However, poor disease status, high EBMT risk score and no combination of UCB were related to relapse (*P* < 0.10, shown in Table 3).

**Table 3:** Univariate analysis of the OS, PFS, TRM, and relapse rate in all patients

Variable	OS		PFS		TRM		Relapse	
	HR (95%CI)	*P* value	HR (95%CI)	*P* value	HR (95%CI)	*P* value	HR (95%CI)	*P* value
Age	1.030 (1.002–1.058)	**0.036**	1.839 (1.037–3.259)	**0.037**	1.692 (0.885–3.237)	0.112	1.706 (0.689–4.223)	0.248
Gender	0.089 (0.340–1.922)	0.631	0.859 (0.390–1.894)	0.707	0.782 (0.314–1.947)	0.597	1.071 (0.309–3.713)	0.914
Height	1.213 (0.512–2.876)	0.660	0.829 (0.371–1.850)	0.646	1.207 (0.487–2.991)	0.685	0.538 (0.138–2.099)	0.373
Weight	1.162 (0.489–2.758)	0.734	1.410 (0.633–3.144)	0.401	0.971 (0.395–2.391)	0.950	2.259 (0.582–8.769)	0.239
Disease status	1.500 (0.957–2.351)	**0.077**	1.527 (1.015–2.295)	**0.042**	1.303 (1.047–1.621)	**0.018**	1.815 (0.951–3.461)	**0.070**
Donor type	0.910 (0.473–1.751)	0.777	1.070 (0.559–2.047)	0.839	0.851 (0.440–1.643)	0.630	1.200 (0.393–3.667)	0.748
Mismatched HLA loci	0.943 (0.561–1.587)	0.827	1.146 (0.731–1.797)	0.552	1.036 (0.609–1.764)	0.896	1.626 (0.849–3.113)	0.143
Blood type	1.179 (0.789–1.761)	0.422	1.166 (0.798–1.704)	0.427	1.070 (0.693–1.651)	0.761	0.911 (0.472–1.761)	0.783
MNC count	1.373 (0.583–3.235)	0.468	1.015 (0.456–2.259)	0.972	1.358 (0.551–3.343)	0.506	0.669 (0.173–2.589)	0.561
CD34 + cells count	0.595 (0.200–1.771)	0.351	0.612 (0.229–1.632)	0.326	0.678 (0.225–2.048)	0.491	0.614 (0.130–2.903)	0.539
EBMT risk score	2.653 (1.366 -5.152)	**0.004**	2.637 (1.438–4.835)	**0.002**	2.383 (1.219–4.662)	**0.011**	3.998 (1.291–12.379)	**0.016**
aGVHD	1.290 (0.548–3.038)	0.560	1.083 (0.494–2.373)	0.843	1.611 (0.648–4.007)	0.305	1.190 (0.344–4.114)	0.783-
cGVHD	0.281 (0.083–0.956)	**0.042**	0.316 (0.108–0.921)	**0.035**	0.319 (0.093–1.096)	**0.070**	0.693 (0.179–2.681)	0.595
Infection	3.887 (1.635–9.239)	**0.002**	3.100 (1.413–6.803)	**0.005**	4.944 (1.944–12.574)	**0.001**	1.922 (0.541–6.827)	0.312-
UCB	0.409 (0.173–0.967)	**0.035**	0.376 (0.171–0.826)	**0.011**	0.409 (0.165–1.009)	**0.045**	0.169 (0.043–0.657)	**0.004**

Bolded variable represented *P* < 0.10

OS, overall survival; PFS, progression-free survival; TRM, transplant-related mortality; HR, hazard ratio; CI, confidence interval; HLA, human leukocyte antigen; MNC, mononuclear cell; EBMT, the European group for blood and marrow transplantation; GVHD, graft versus host disease. Factors with *P* > 0.10 were excluded

### Multivariate analysis

In multivariate analysis (Table 4), the combination of UCB units (HR 0.331; 95% CI 0.130–0.843; *P* = 0.020) was an independent factor for improving OS. Infection (HR 2.964, 95% CI 1.172–7.496; *P* = 0.022) and cGVHD (HR 0.199; 95% CI 0.054–0.729; *P* = 0.015) were independent factors associated with poor OS. Meanwhile, the combination of UCB units (HR 0.338; 95% CI 0.144–0.795; *P* = 0.013) was an independent factor for improving PFS. However, cGVHD (HR 0.232; 95% CI 0.074–0.72; *P* = 0.012) was an independent factor associated with poor PFS. For TRM, the combination of UCB units (HR 0.329; 95% CI 0.121–0.889; *P* = 0.028) was an independent factor for reducing TRM. Infection (HR 4.104, 95% CI 1.506–11.186; *P* = 0.006) and cGVHD (HR 0.228; 95% CI 0.061–0.850; *P* = 0.028) were independent factors associated with increased TRM. The only independent factor for relapse was the combination of UCB units (HR 0.243; 95% CI 0.061–0.973; *P* = 0.046), which could reduce relapse.

**Table 4:** Multivariate analysis of the OS, PFS, TRM, and relapse rate in all patients

Variable	OS		PFS		TRM		Relapse	
	HR (95% CI)	*P* value	HR (95% CI)	*P* value	HR (95% CI)	*P* value	HR (95% CI)	*P* value
Age	0.967 (0.444–2.105)	0.932	0.861 (0.429–1.729)	0.675	–	–	–	–
Disease status	1.187 (0.692–2.036)	0.533	1.281 (0.779–2.105)	0.329	1.090 (0.621–1.914)	0.764	1.254 (0.606–2.595)	0.542
EBMT risk score	1.804 (0.719–4.529)	0.209	1.876 (0.819–4.297)	0.137	1.560 (0.725–3.357)	0.256	2.816 (0.821–9.660)	0.100
cGVHD	0.199 (0.054–0.729)	**0.015**	0.232 (0.074–0.722)	**0.012**	0.228 (0.061–0.850)	**0.028**	–	–
Infection	2.964 (1.172–7.496)	**0.022**	2.165 (0.928–5.053)	0.074	4.104 (1.506–11.186)	**0.006**	–	–
UCB	0.331 (0.130–0.843)	**0.020**	0.338 (0.144–0.795)	**0.013**	0.329 (0.121–0.889)	**0.028**	0.243 (0.061–0.973)	**0.046**

Bolded variable represented *P* < 0.05

OS, overall survival; PFS, progression-free survival; TRM, transplant-related mortality; HR, hazard ratio; CI, confidence interval; EBMT, the European group for blood and marrow transplantation; GVHD, graft versus host disease. Factors with *P* > 0.05 were excluded

## Discussion

Until recently, improving the outcomes of haplo-HSCT and enhancing the GVL effect without aggravating GVHD effects were the main concerns. The biology of grafts was the first thing needing to be known. Currently, grafts for transplantation mainly include peripheral blood, bone marrow and UCB hematopoietic cells. The grafts of classical haplo-HSCT did not include UCB units. Previous laboratory research showed that UCB was a rich source of hematopoietic stem (HSCs) and progenitor (HPC) cells [16]. Studies also confirmed that the hematopoietic reconstitution capacity of UCB-derived HSCs in immune-deficient mice was superior to that of adult marrow cells in vivo [17]. Furthermore, UCB possesses great proliferation and expansion potential [18]. Therefore, umbilical cord blood was also used as an HSCT graft. Initially, UCB transplantation (UCBT) was used in children and achieved satisfactory outcomes [19]. Because UCB has few CD34^+^ cells, new approaches, such as the use of double UCBT (dUCBT), have been used in adult patients to avoid a prolonged delay in immune reconstitution [20]. The progress of UCBT was also summarized in a review [21]. In a previous study of UCBT, the incidence of severe GVHD was found to be lower than that of HLA-matched HSCT, especially HLA-matched unrelated donor HSCT [22]. The cause of GVHD was possibly attributable to the reactivity of donor T cells with recipient minor histocompatibility antigens. The reason why UCB grafts could reduce GVHD after UCBT is mainly as follows. First, UCB T lymphocytes are typically CD45RA^+^ naïve T cells with low levels of activation markers [23]. Second, altered recognition of recipient self-antigens by UCB donor T cells may result upon interaction with the recipient’s antigen presenting cells (APC) [24]. Third, there is a limited response of these naïve donor T cells activated by the recipient alloantigen. In primary mixed lymphocyte culture, UCB T cells demonstrate proliferative responses to allogeneic stimulation but less cytotoxic effector function, less proliferation and greater activation-induced cell death (AICD) [25]. Fourth, these changes result in impaired cytokine production, limited cellular activation and lack of clonal expansion of alloreactive T cells. UCB immune tolerance includes altered toll-like receptor and adhesion molecule expression on donor graft APCs [26]. Studies have also suggested that UCB graft T cells display reduced expression of nuclear factor of activated T cells-1 (NFAT1), which may be one important molecular mechanism underlying their reduced capacity for cytokine production [27, 28]. Taken together, the lower incidence and severity of GVHD found in UCB recipients is a direct consequence of the reduced proliferation, cytokine production, and cytotoxicity to alloantigens displayed by UCB lymphocytes. On the other hand, because of its biology, UCB permits a greater degree of HLA mismatching with an acceptable incidence of GVHD, without compromising the GVL effect. In addition, significant immunity against leukemia and viral antigens was provided by lymphocytes in the UCB graft. As previously mentioned, dUCBT improved the hematopoietic reconstitution in adult patients. Furthermore, studies also showed that dUCBT enhanced the GVL effect through the graft-versus-graft (GVG) effect because CD4^+^ T cells from the predominant UCB could rapidly reject nonengrafting UCB [29]. The enhanced GVL effect through the GVG effect during dUCBT is also mediated by specific CD8^+^ T-cell responses [30]. Another important concern for UCBT is hematopoietic and immune reconstitution. The TNC and CD34^+^ cells in the UCB unit were limited. However, UCB had a less mature phenotype of CD34^+^ progenitors compared to adult marrow and peripheral blood grafts, which might have a higher proliferation potential than adult CD34^+^ cells [31]. Taken together, the UCB unit, as one of the grafts for allo-HSCT, could reduce the GVHD effect, enhance the GVL effect and promote engraftment. The UCB unit as a transplantation graft might be considered a supplement for haploidentical HSCT.

In the present cohort study, we compared the outcomes between UCB-haplo HSCT and haplo HSCT. The result confirmed our postulation that the third-party UCB unit could enhance the GVL effect for reducing the risk of relapse and elevating the OS and PFS in haplo-HSCT. The risks of TRM and relapse were significantly decreased in the UCB-haplo group. Disease relapse was the primary cause of transplantation-related failure and death. In our study, the cumulative incidence of relapse was also decreased in the UCB-haplo group. This result was consistent with previous studies on the combination of UCB units in haplo-UCBT [8, 32]. GVHD was also a concern in HSCT. We analyzed aGVHD, including II-IV aGVHD and III-IV aGVHD, and cGVHD, including extensive cGVHD, in both groups. The results showed that the combination of third-party UCB did not increase any GVHDs. The results were also consistent with previous studies [8, 32]. As previously reported, UCBT might cause an increase in infection [33], especially CMV and EBV infection [34, 35]. In our study, the 100-day cumulative incidences of CMV viremia and EBV viremia were not significantly different between the UCB-haplo group and the haplo group. This result was consistent with that of Liu et al. [36]. However, they found that the combination of haploidentical and UCB HSCT resulted in rapid engraftment, yielding a different conclusion from our study. Our study showed that there were no significant differences between the UCB-haplo group and the haplo group. However, the transplantation regimen was different from that used by Liu et al. with reduced-intensity conditioning and our study with the “Beijing protocol,” which was a myeloablative conditioning regimen.

The present study showed that combining the third-party UCB unit with haploid grafts to perform UCB-haplo HSCT achieved better outcomes due to the GVL effect without increasing GVHD or infections. To determine the function of the UCB unit in HSCT, the comparison between single-UCBT and dUCBT studies was also reviewed. The outcomes of previous studies reached conflicting conclusions. Verneris et al. performed a randomized study in 2009 that found that dUCBT could decrease the risk of relapse, indicating the enhancement of the GVL effect, but increased the II–IV aGVHD effect [37]. Gérard Michel et al. found that dUCBT did not decrease transplantation strategy failure and even caused extensive cGVHD more frequently through a prospective randomized study in 2016 [38]. The conflicting results might be due to the mismatch between UCB units and receipts. Tozatto-Maio et al. found that a lower number of HLA mismatches with the recipient was indicated in dUCBT for acute leukemia patients [39]. Wang et al. compared the outcomes between cord-haplo and haplo-HSCT in refractory acute leukemia and established a mutual haploidentical donor mismatched antigen (MHMA) algorithm. The results showed that MHMA influenced both relapse and TRM in patients in the cord-haplo group. Patients with 1 MHMA had the most favorable PFS rate [8]. Lamers et al. suggest that cytotoxicity exerted by CD4^+^ T cells from the predominant UCB toward HLA class II alleles drives the rapid rejection of the nonengrafting UCB, whose alloreactive effect might also contribute to the GVL effect. In our study, we selected the UCB units with the strategy previously mentioned. We reviewed our data and found that the selection of mismatched UCB units had no significant effect on OS, PFS, relapse or TRM. First, the comparison between UCB units and patients on HLA-A, -B and -DR was performed as Gérard Michel et al. mentioned [38]. The results showed that more mismatched loci might predict longer PFS and OS and a lower risk of TRM, but the difference was not significant. There was nearly the same risk of relapse between groups in this algorithm. Second, the comparison was performed with the MHMA algorithm as Wang et al. established [8]. More mismatched loci represented longer PFS and a lower risk of relapse, but there was also no significant difference. There was no difference in TRM or OS between the groups. Third, HLA class II allele mismatch comparisons were performed as Lamers et al. suggested [29]. Furthermore, mismatches of HLA-A, -B and -DR comparisons were also calculated between UCB units and haploid donors. The results showed that more mismatched loci indicated better outcomes, while there was also no significant difference. The HLA-C and HLA-DQ loci were also compared between groups. The result also provided clues to us that the more mismatched loci there were, the better the outcomes, also without any significant difference.

There were several limitations in the present study. The result came from a single center with limited patients. However, we found that the combination of UCB units and haploid grafts significantly improved patient outcomes during HSCT. The conclusions remain to be validated in further independent and more extensive studies. We found clinical results that indicated that third-party UCB could enhance the prognosis, while there are still many experimental studies to confirm our conclusion.

## Conclusion

Above all, the combination of third-party UCB units in haplo-HSCT increased the GVL effect without enhancing the GVHD effect. This result might be attributed to the biology of UCB and the GVG effect between the two grafts.

## Data Availability

The data was availability. All the data could ask for corresponding author to provide by email.

## Abbreviations

haplo-HSCT: Haploidentical hematopoietic stem cell transplantation
GVL: Graft versus leukemia
GVHD: Graft versus host disease
UCB-haplo HSCT: Unrelated umbilical cord blood
OS: Overall survival
PFS: Progression-free survival
TRM: Transplantation-related mortality
allo-HSCT: Allogeneic hematopoietic stem cell transplantation
HLA: Human leukocyte antigen
AML: Acute myeloid leukemia
MDS: Myelodysplastic syndrome
AA: Aplastic anemia
GVL: Graft versus leukemia
UCB: Umbilical cord blood
STR-PCR: Short tandem repeat polymerase chain reaction
HSCs: Hematopoietic stem cells
HPC: Progenitor cells
UCBT: UCB transplantation
dUCBT: Double UCBT
APC: Antigen presenting cells
AICD: Activation-induced cell death
NFAT1: Nuclear factor of activated T cells1
GVG: Graft-versus-graft
MHMA: Mutual haploidentical donor mismatched antigen
